# Genetic Loci Associated with Resistance to Zucchini Yellow Mosaic Virus in Squash

**DOI:** 10.3390/plants10091935

**Published:** 2021-09-17

**Authors:** Swati Shrestha, Vincent Njung’e Michael, Yuqing Fu, Geoffrey Meru

**Affiliations:** Horticultural Sciences Department, Tropical Research and Education Center, University of Florida, Homestead, FL 33031, USA; s.shrestha@ufl.edu (S.S.); michael.vn@ufl.edu (V.N.M.); yuqingf@ufl.edu (Y.F.)

**Keywords:** QTL-seq, breeding, disease resistance, potyviruses, quantitative trait loci, marker-assisted selection, whole genome resequencing, *Cucurbita*

## Abstract

Zucchini Yellow Mosaic Virus (ZYMV) is an aphid-transmitted potyvirus that causes severe yield losses in squash (*Cucurbita moschata*) production worldwide. Development of resistant cultivars using traditional breeding approaches relies on rigorous and resource-intensive phenotypic assays. QTL-seq, a whole genome re-sequencing based bulked segregant analysis, is a powerful tool for mapping quantitative trait loci (QTL) in crop plants. In the current study, the QTL-seq approach was used to identify genetic loci associated with ZYMV resistance in an F_2_ population (n = 174) derived from a cross between Nigerian Local (resistant) and Butterbush (susceptible). Whole genome re-sequencing of the parents and bulks of resistant and susceptible F_2_ progeny revealed a mapping rate between 94.04% and 98.76%, and a final effective mapping depth ranging from 81.77 to 101.73 across samples. QTL-seq analysis identified four QTLs significantly (*p* < 0.05) associated with ZYMV resistance on chromosome 2 (*QtlZYMV-C02*), 4 (*QtlZYMV-C04*), 8 (*QtlZYMV-C08*) and 20 (*QtlZYMV-C20*). Seven markers within the QTL intervals were tested for association with ZYMV resistance in the entire F_2_ population. For *QtlZYMV-C08*, one single nucleotide polymorphism (SNP) marker (KASP-6) was found to be significantly (*p* < 0.05) associated with ZYMV resistance, while two SNPs (KASP-1 and KASP-3) and an indel (Indel-2) marker were linked to resistance within *QtlZYMV-C20*. KASP-3 and KASP-6 are non-synonymous SNPs leading to amino acid substitutions in candidate disease resistant gene homologs on chromosomes 20 (*CmoCh20G003040.1*) and 8 (*CmoCh08G007140.1*), respectively. Identification of QTL and SNP markers associated with ZYMV resistance will facilitate marker-assisted selection for ZYMV resistance in squash.

## 1. Introduction

The major cultivated species of *Cucurbita* (*C. pepo*, *C. moschata*, *C. maxima*, *C. argyrosperma* and *C. ficifolia*) originated from Central and South America and include cultivars of squash and pumpkin, which are economically important worldwide [1,2]. Squashes and pumpkins are highly valued for culinary, seed-snacking, seed-oil and pharmaceutical purposes [3,4,5,6]. In 2020, 47,186 hectares of land were dedicated to squash and pumpkin production in the U.S., yielding 1,158,214 tons of the crop, which was valued at approximately USD 412 million [7].

Plant diseases caused by viruses are among the major limiting factors in *Cucurbita* production worldwide [8]. More than twenty viruses are reported to infect *Cucurbita* spp., among which Zucchini Yellow Mosaic Virus (ZYMV), an aphid-transmitted potyvirus, is one of the most destructive, affecting both yield and fruit quality [9,10]. Symptoms caused by ZYMV in *Cucurbita* are similar to those elicited by other mosaic viruses, and include yellowing, mottling and blistering of leaves, fruit distortion, stunting and reduced seed set [11]. Management of ZYMV depends heavily on the suppression of insect-vector (aphid) population using insecticides but is not wholly effective especially under high aphid pressure [12]. Development and deployment of virus-resistant *Cucurbita* cultivars is the most effective strategy for mitigating yield losses caused by ZYMV, thus a major goal for squash breeders [12,13].

Munger and Provvidenti [14] identified a natural source of resistance to ZYMV in ‘Nigerian Local’, a landrace of *C. moschata* from Nigeria. Resistance in Nigerian Local is conferred by two genes, *Zym-0* and *Zym-4*, with the former conferring resistance independently, and the latter in a complementary fashion with a recessive gene *zym-5* from Waltham butternut [11,15]. The resistance alleles for ZYMV in Nigerian Local have been widely used in breeding through phenotyping-based selection methods [15] and numerous resistant commercial cultivars are currently available in the market [13]. However, breeding for disease resistance through phenotypic selection is rigorous and resource intensive, and is often complicated by genotype by environment interaction. As an alternative, molecular tagging of alleles linked to ZYMV resistance can assist identification of desired resistant individuals in segregating populations through marker-assisted selection (MAS). Using bulk segregant analysis (BSA), Pachner et al. [16] identified a single simple sequence repeat (SSR) marker (*TGM-Zym-0*) associated with resistance to ZYMV in an F_2_ population derived from Nigerian Local × Waltham butternut (susceptible). However, the sequence and genomic location of *TGM-Zym-0* is not publicly available, and its routine application in MAS may be prohibitive for small breeding programs due to the high cost of fluorescently labeled SSR assays and capillary-based electrophoresis [17]. In addition, no quantitative trait loci (QTL) or candidate genes are currently described for ZYMV resistance in Nigerian Local that would facilitate marker development and characterization of the mechanisms underlying the trait.

The small genome size of *C. moschata* (approximately 372 Mb) and the recently available reference genome [18] for the species provides an opportunity for marker discovery and genetic mapping of important traits. QTL-seq is a rapid and powerful technique for genetic mapping that combines BSA with whole-genome resequencing [19] to identify genomic regions significantly associated with traits of interest in segregating populations. QTL-seq has been successfully employed to identify QTL associated with economically important traits in both agronomic and horticultural crops including chickpea [20], rice [19,21], cucumber [22], watermelon [23,24], Broccoli [25,26] and squash [27].

The aim of the current study was to identify QTL, molecular markers and candidate genes associated with ZYMV resistance in Nigerian Local using the QTL-seq approach. Deployment of molecular markers linked to ZYMV resistance in the breeding program would facilitate MAS, thus accelerating the release of resistant commercial squash cultivars.

## 2. Materials and Methods

### 2.1. Plant Material and ZYMV Inoculum

A cross between Nigerian Local (resistant) and Butterbush (susceptible) was made in the greenhouse and a single F_1_ plant was selfed to yield an F_2_ population (n = 174). For inoculum preparation, plants of Yellow Crookneck, a susceptible summer squash cultivar (*C. pepo*), were inoculated with a virulent isolate of ZYMV-FL (provided by Dr. Jane Polston, University of Florida), and maintained in insect-proof (160 µm aperture) cages (Megaview Science, Talchung, Taiwan). The isolate was confirmed as ZYMV using a ZYMV-specific immunostrips and enzyme linked immunosorbent assay (ELISA) following manufacturer’s instructions (Agdia Inc., Elkhart, IN, USA).

### 2.2. Phenotyping

Seeds of parents (n = 10) and F_2_ (n = 174) were sown in 4-inch pots containing Proline C/B growing mix (Jolly Gardener, Quakertown, PA, USA) amended with 14N-4.2P-11.6K controlled-release fertilizer (Osmocote; Scotts, Marysville, OH, USA). Ten seeds each of two *C. pepo* summer squash cultivars (Yellow Crookneck and Early Prolific) were also included in the experiment as susceptible checks. At the second true-leaf stage, the cotyledons and the oldest leaf were lightly dusted with silicon carbide powder (Thermo Fisher Scientific, Waltham, MA, USA) and inoculated with ZYMV. The inoculum was prepared by macerating fresh-leaves of ZYMV-infected Yellow Crookneck cultivar in 0.02 M phosphate buffer (pH 7.0) at a 1:5 (*w*/*v*) tissue-to-buffer ratio. Disease severity (DS) data was collected at 14, 21 and 28 days after inoculation (DAI). Data was recorded based on a presence/absence rating scale, where plants exhibiting ZYMV symptoms (yellowing, mottling, and blistering of the leaves) were given a score of 1, while asymptomatic plants were assigned a score of 0. Plants with a score of 0 and 1 were regarded as resistant and susceptible, respectively. Ten random plants each from the resistant and susceptible groups were selected for DNA extraction, whole genome resequencing, and subsequent QTL analysis.

### 2.3. DNA Extraction and Whole Genome Re-Sequencing

DNA extraction from ten resistant (DS = 0) and ten susceptible (DS = 1) F_2_ progeny and the parents was carried out from emerging first true leaf using the FavorPrep Plant DNA kit (Favorgen Biotech Corp, Ping-Tung, Taiwan) according to the manufacturer’s instructions. DNA concentration was determined using NanoDrop 8000 (Thermo Fisher Scientific, Waltham, MA, USA) and equal amounts (500 ng) from each of the 10 individuals constituting a bulk were pooled. Library (2 × 150 paired-end) construction and whole genome re-sequencing of the parents and the two bulks was performed on the Illumina HiSeq X (Illumina, Inc., San Diego, CA, USA) at the BGI sequencing center (Shenzhen, Guangdong, China).

### 2.4. Construction of Consensus Sequence and QTL-Seq Analysis

To obtain a consensus reference fasta file, cleaned short raw reads of both the parents (Nigerian Local and Butterbush) were aligned to reference genome of *C.* *moschata* (Rifu) [18] using BWA-MEM [28]. Consensus reference fasta files were built using SAMtools mpileup by replacing *C. moschata* (Rifu) reference alleles with the respective parent alleles across the genome [18,28]. Sequence reads from the resistant and susceptible bulks were then mapped onto the consensus fasta files following standard bioinformatics procedures [29]. Briefly, aligned reads of resistant and susceptible bulks were converted from SAM to BAM files using SAM tools 1.9, then Picard 2.19.1 was used to replace all the read groups in the input file with a single new read group and assign all the read group to this new read group in the output BAM files [29,30]. Duplicate reads were removed using Picard 2.19.1, and Haplotype caller of GATK 4.0.5.0 was used for local realignment and SNPs/INDELs calling using consensus sequence as reference [31]. The called variants from both bulks were then combined using Genotype GVCFs to obtain a single raw variant VCF file, which was subsequently converted into table format for QTL analysis in QTLseqr [18,31,32]. SNPs were filtered in QTLseqr with minimum depth of 100, maximum depth of 500, minimum GQ of 99. The *runQTLseqAnalysis* and *runGprimeAnalysis* functions were implemented in QTLseqr to detect QTL associated with ZYMV resistance [33,34]. At each SNP, the ∆SNP-index was calculated by determining the difference between SNP-index of resistant and susceptible bulks [19,33]. G’ statistic for each SNP was calculated from the observed and expected allele depths, followed by smoothing through tricube smoothing kernel [34]. G’ statistics at a false discovery rate of 0.01 and ∆SNP-index at 95% confidence interval were calculated over 1 Mb windows.

### 2.5. Marker Test and Candidate Genes Identification

To validate the identified QTL, 12 indel and 14 SNP markers within candidate QTL intervals were genotyped in the F_2_ population (Table S1). Genomic regions flanking the target markers were extracted from the *C. moschata* (Rifu) genome available through the Cucurbit Genomics Database [35]. Indel markers were designed using Primer3Plus [36] and PCR assays were carried out in 15-μL reactions containing 25 ng of template DNA, 0.4 μM each of forward and reverse primers, and 1X PROMEGA Colorless GoTaq^®^ master mix (Promega, Madison, WI, USA). Amplification was performed on a SimpiAmp thermalcycler (Applied Biosystems, Foster City, CA, USA) using an initial 3-min denaturation step at 95 °C, followed by 35 cycles of 15 s at 95 °C, 20 s at 52 °C, and 30 s at 72 °C. Amplicon sizes were determined on a 2% *w*/*v* agarose gel. For SNPs, oligonucleotides for Kompetitive allele specific PCR (KASP) assays were designed using BatchPrimer3 software [37], and the assays were performed in 10-μL reactions containing 5-μL of 2X low rox KASP master mix (LGC Genomics LLC, Teddington, UK), 0.16 μL each of forward primers (10 μM), 0.41 μL of reverse primer, 2 μL of genomic DNA (50 ng/μL) and 2.27 μL of H2O. The PCR conditions consisted of an initial incubation at 94 °C for 15 min, a touchdown PCR at 94 °C for 20 s, 61 °C for 60 s, with a 0.6 °C decrease per cycle for 10 cycles, followed by 26 cycles of 94 °C for 20 s and 55 °C for 60 s. Fluorescent end-point readings and cluster calling were performed using LightCycler^®^ 480 Instrument II (Roche Life Sciences, Penzberg, Germany). The Kruskal–Wallis test (*p* ≤ 0.05) was used to test the association of markers with ZYMV resistance in the F_2_ population using R 1.3.1 statistical software [38]. Markers that showed significant association with ZYMV resistance were further validated by testing in a backcross (BC_1_F_1_) population developed by crossing F_1_ (Butterbush × Nigerian Local) with Butterbush as recurrent parent. Furthermore, utility of the markers was tested in a set of ZYMV-susceptible commercial *C. pepo* (Yellow Crookneck and Early Prolific) and *C. moschata* (Waltham butternut) cultivars.

Candidate disease resistant genes within each significant QTL interval were identified by querying the gff3 and GO reference files from Cucurbit Genomics Database against the PRGdb database (Plant Resistance gene Database, http://prgdb.crg.eu/, accessed on 14 October 2020) [39]. Effect of significant SNPs on predicted amino acids sequence was investigated using *C. moschata* (Rifu) genomic resources [35].

### 2.6. Synteny with Cucurbit Crops

To evaluate if the significant QTL regions identified in *C. moschata* were syntenic with ZYMV resistance loci in cucumber (*Cucumis sativus*), melon (*Cucumis melo*) and watermelon (*Citrullus lanatus*), analysis was performed using “Synteny Viewer” feature of Cucurbit Genomics Database) [35]. Briefly, the blastp function was used to query protein sequences within significant QTL regions against protein databases for cucumber, melon and watermelon, and then synteny blocks were identified for resulting alignments using MCScanX [40].

## 3. Results

### 3.1. Phenotypic Data

**Figure 1:**
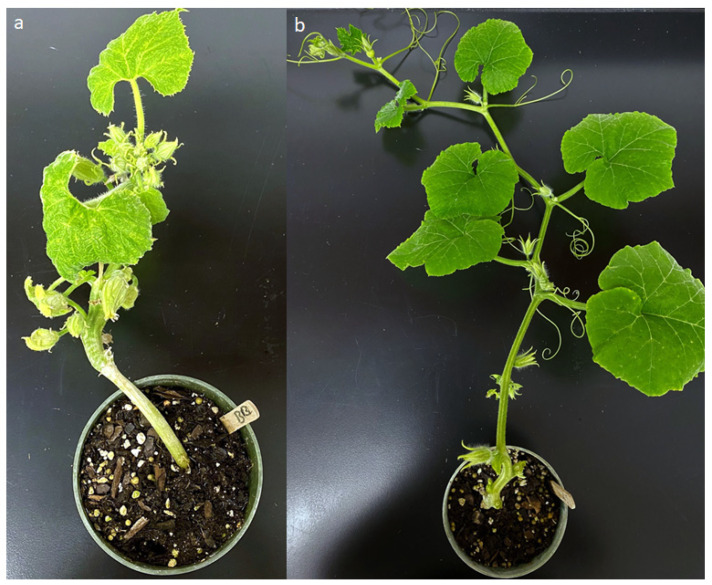
Symptom severity in (**a**) Butterbush (leaf yellowing, mottling and stunted growth) and (**b**) Nigerian Local (asymptomatic) at 28 days after inoculation with Zucchini Yellow Mosaic Virus.

At 28 DAI, Nigerian Local plants exhibited high resistance to ZYMV and were asymptomatic (mean DS = 0), whereas the susceptible parent (Butterbush) showed leaf yellowing, mottling and stunted growth (mean DS = 1) (Figure 1). The two susceptible *C. pepo* cultivars (Yellow Crookneck and Early Prolific) were also highly susceptible. Among F_2_ individuals, 55% were resistant and 45% were susceptible based on the absence/presence ZYMV rating scale used in the current study.

### 3.2. QTL Analysis

Mapping rate across the samples varied from 94.04% to 98.76% with final effective mapping depth ranging from 81.77 to 101.73 (Table 1).

**Figure 2:**
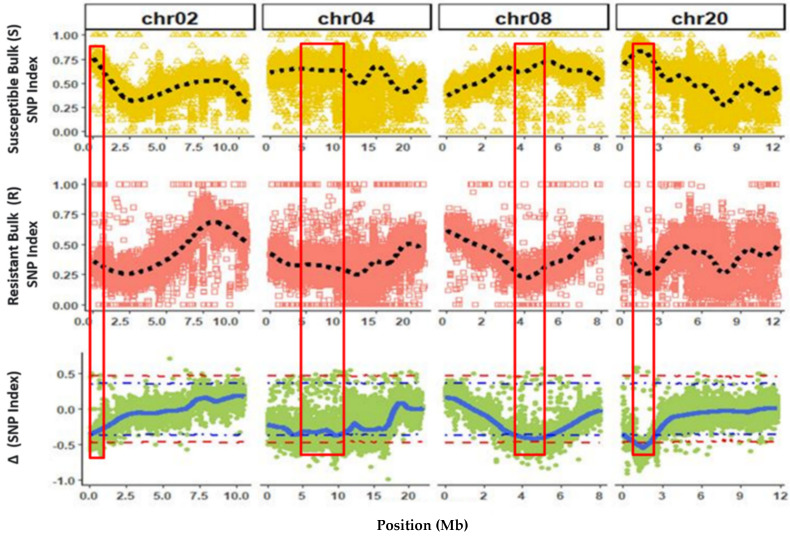
Quantitative trait loci (regions within red columns) associated with Zucchini Yellow Mosaic Virus resistance in Nigerian Local accession (*Cucurbita moschata*) on chromosome (chr) 02, 04, 08 and 20 using Butterbush as consensus reference genome. The dotted black lines represent smoothed conditional mean of SNP index for Susceptible (S) and Resistant (R) bulks, while the solid blue line represents the tricube ΔSNP for the ΔSNP index (R SNP index − S SNP index). The purple and red dotted lines in the ΔSNP index plot are the 95% and 99% confidence intervals for the regions, respectively.

Alignment of bulks onto the consensus reference genome for Butterbush and Nigerian Local revealed 1,907,191 and 1,916,964 SNP’s, respectively. QTL-seq analysis identified four QTLs significantly associated with ZYMV resistance on chromosome 2 (*QtlZYMV-C02*), 4 (*QtlZYMV-C04*), 8 (*QtlZYMV-C08*), and 20 (*QtlZYMV-C20*) (Figure 2, Figure S1).

There was slight variation in the significant interval for each region depending on the parental consensus reference genome used; however, the position of the highest ΔSNP-index was consistent across the QTL, with the exception of *QtlZYMV-C02* where a 1.7 kb shift in position occurred (Table 2). The intervals for the detected QTL were smallest in *QtlZYMV-C02* (4.7 kb) and largest for *QtlZYMV-C20* (2.46 Mb), with Butterbush as parental consensus reference genome.

Similar QTLs were detected using both ΔSNP-index (95% significance level) and G’ analyses (0.01 false discovery rate) approaches (Figure S2).

### 3.3. Marker Test and Candidate Genes Associated with ZYMV Resistance

The parents and the F_2_ individuals comprising the resistant and susceptible bulks were initially genotyped with 12 indel and 14 SNP markers (Table S1). Seven of these markers (1 indel and 6 SNPs) were polymorphic between the parents, and distinguished susceptible and resistant individuals in the bulks, thus were genotyped in the entire F_2_ population consisting of 174 individuals. Kruskal–Wallis test indicated that one SNP on chromosome 8 (KASP-6), two SNPs (KASP-1 and KASP-3) and one indel (Indel-2) on chromosome 20 were significantly associated (*p* < 0.05) with resistance to ZYMV (Table 3; Figure S3). Indel 2 amplified a 245 bp fragment in the resistant individuals, but a null allele in the susceptible individuals (Figure S3). Utility of the significant markers was confirmed (*p*< 0.05) in ZYMV susceptible cultivars (Yellow Crookneck, Early Prolific, and Waltham butternut) and a BC_1_F_1_ population (Table S2). No markers significantly associated with resistance to ZYMV were detected for *QtlZYMV-C02* and *QtlZYMV-C04* in the F_2_ population.

A scan of the four QTL intervals revealed 922 genes: 1 within *QtlZYMV-C02*, 123 within *QtlZYMV-C04*, 373 within *QtlZYMV-C08*, and 520 within *QtlZYMV-C20.* Among these, 37 were annotated as resistant gene homologs: 7 nucleotide-binding sites leucine-rich repeat, 10 serine/threonine protein kinase, 3 protein phosphatases, 12 receptor-like kinases, 4 NB-ARC protein, and 1 stress protein (Universal stress protein A) (Table S3). SNP markers significantly associated with ZYMV resistance (KASP-1, KASP-3 and KASP-6) were further investigated for their impact on predicted amino acids sequence. KASP-1 (*QtlZYMV-C20*) is a synonymous point mutation (C/A at 1,491,656 bp) within an exon for *CmoCh20G003050.1* and does not impact predicted amino acid sequence. However, KASP-3 (*QtlZYMV-C20*) is non-synonymous point mutation (T/A at 1,486,889 bp) resulting in amino acid substitution from lysine (susceptible parent) to aspartate (resistant parent) in *CmoCh20G003040.1* gene. Similarly, KASP-6 (*QtlZYMV-C08*) is a non-synonymous point mutation (A/C at 4,497,085 bp) in *CmoCh08G007140.1* gene leading to amino acid substitution from phenylalanine (susceptible parent) to leucine (resistant parent).

### 3.4. Synteny with Cucurbits

**Figure 3:**
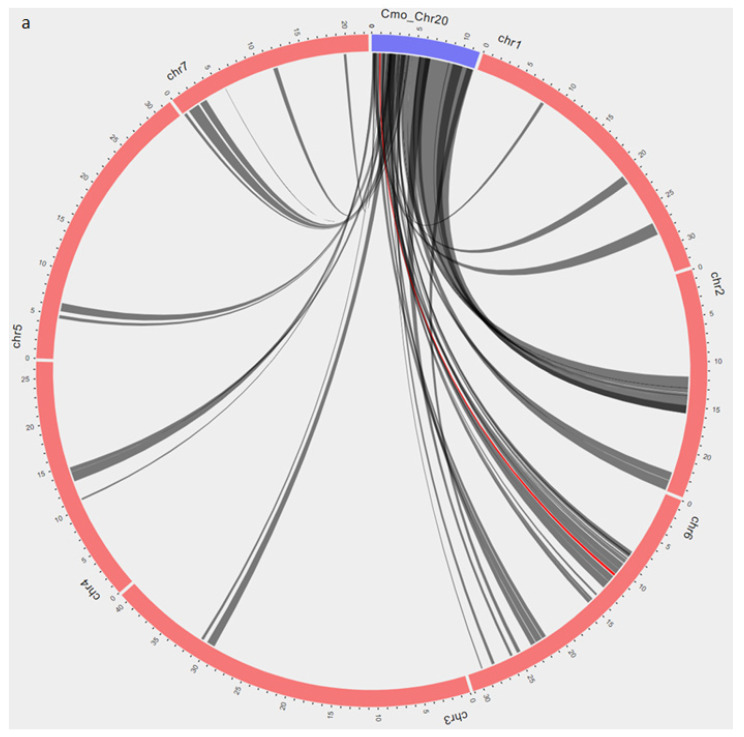

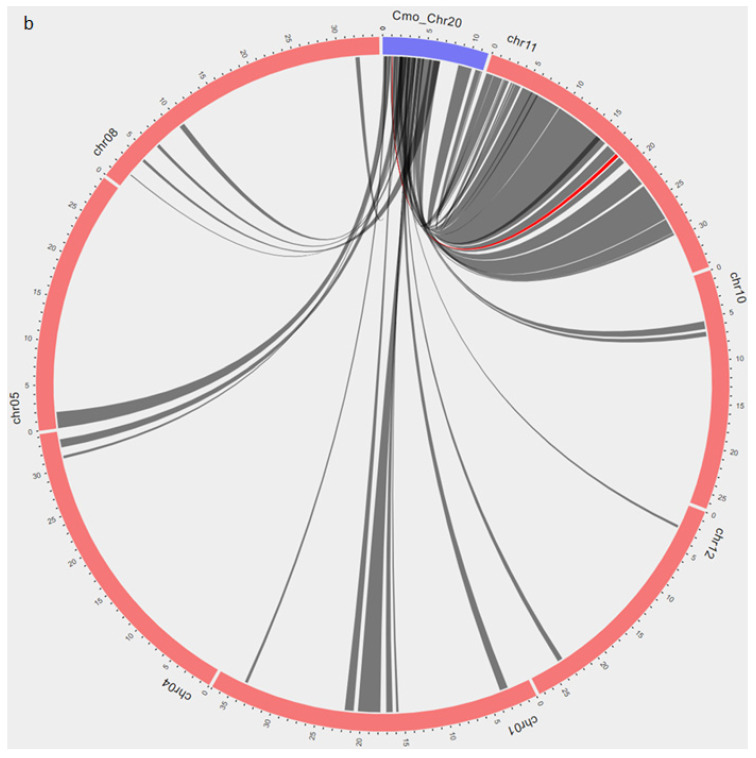
Synteny blocks are displayed in a Circos plot between (**a**) squash (*C. moschata*) and cucumber (*Cucumis sativus*) and (**b**) squash and melon (*Cucumis melo*). Blue arcs represent chromosome 20 of *C. moschata* (Rifu). Red arcs represent chromosomes of cucumber (**a**) and melon (**b**), respectively. Dark gray lines indicate syntenic regions between squash and the two genomes. Red lines show syntenic blocks associated with ZYMV resistance (**a**) between chromosome 20 of *C. moschata* and chromosome 6 of cucumber and (**b**) between chromosome 20 of *C. moschata* and chromosome 11 of melon.

In cucumber, a single recessive gene (*zym*) mapped on chromosome 6 confers resistance to ZYMV [41,42]. The genomic location of *zym* locus extends from 10,725,863–10,967,931 bp and shares a syntenic block with *QtlZYMV-C20* resistance loci in *C. moschata* on chromosome 20 (897,253–1,010,607 bp) (Figure 3a). In melon, a dominant gene on chromosome 11 (*MELO3C013571.2.1*) confers resistance to ZYMV [43,44]. QTL harboring *MELO3C013571.2.1* (17,975,050–18,397,354 bp) is syntenic to genomic region for *QtlZYMV-C20* (1,092,371–1,152,668 bp) in squash (Figure 3b). No syntenic blocks were found between *QtlZYMV-C20* and ZYMV resistance locus in watermelon. Similarly, there were no syntenic blocks between *QtlZYMV-C02*, *QtlZYMV-C04* and *QtlZYMV-C08* with ZYMV resistance loci in cucumber, melon and watermelon.

## 4. Discussion

Although the genetics of resistance to ZYMV in Nigerian Local accession have been previously described [11], the QTL and candidate genes associated with the resistance are currently unknown. On the contrary, genomic regions and candidate genes associated with resistance to ZYMV in other major cucurbit crops including watermelon, melon and cucumber have been accomplished [41,42,43,44,45]. Pachner et al. [16] used BSA to identify a single SSR marker (*TGM-Zym-0*) associated with resistance to ZYMV in Nigerian Local, however, the sequence and genomic location of *TGM-Zym-0* is not publicly available. In the current study, four QTLs associated with ZYMV resistance in Nigerian Local were identified on chromosome 2 (*QtlZYMV-C02*), 4 (*QtlZYMV-C04*), 8 (*QtlZYMV-C08*), and 20 (*QtlZYMV-C20*). Inheritance studies in Nigerian Local indicate that at least three genes (*Zym-0*, *Zym-4* and *zym-5*) confer resistance to ZYMV [11,15]. Our results suggest that *QtlZYMV-C20* and *QtlZYMV-C08* may correspond to *Zym-0* and *Zym-4*, respectively, due to their significant association with ZYMV resistance in the F_2_ population. On the contrary, no markers were significantly associated with resistance for *QtlZYMV-C02* and *QtlZYMV*-C04, indicating that the two QTLs are of minor effect towards ZYMV resistance in Nigerian Local.

The four markers (Indel-2, KASP-1, KASP-3 and KASP-6) identified in the current study are good candidates for MAS in squash breeding programs targeting ZYMV resistance. The indel marker linked to *QtlZYMV-C20* can be easily assayed using standard agarose gel electrophoresis, thus is affordable for small breeding programs. On the other hand, high-throughput genotyping of the three significant SNP markers can be accomplished through KASP assays on 384-well plate format or other genotyping platforms.

Scanning of the *C. moschata* cv. Rifu reference genome revealed that Indel-2 was located 1.6 kb upstream of *CmoCh20G003040.1* gene, whereas KASP-3 (SNP T/A) was located on exon 8 of the same gene. *CmoCh20G003040.1* belongs to the protein kinase family and contains a serine-threonine/tyrosine-protein kinase domain. Protein kinases are key components in plant signal transduction and induction of transcriptional activation in response to pathogen attack [46]. Swiss-Prot analysis of *CmoCh20G003040.1* amino acid sequence showed similarity with *Arabidopsis thaliana* Serine/threonine-protein kinase EDR1 (E-value = 3.5 × 10^−46^), which is involved in regulation of defense response to fungus, bacterium, and water deprivation [47,48]. High homology among these protein sequences indicates that *CmoCh20G003040.1* is a good candidate causal gene for ZYMV resistance in *C. moschata.* KASP-1 (SNP C/A) and KASP-6 (SNP A/C) markers are within exon 3 and exon 4 of *CmoCh20G003050.1* and *CmoCh08G007140.1* gene homologs on chromosome 20 and 8, respectively. The homolog *CmoCh20G003050.1* is an RNA helicase associated with RNA processing, which shares significant homology with DEAD-box ATP-dependent RNA helicase 36 of *A. thaliana* (E-value = 2.2 × 10^−10^). On the other hand, *CmoCh08G007140.1* is putative receptor kinase with leucine-rich repeat domain and protein kinase activity and shares significant similarity with probable leucine-rich repeat receptor-like serine/threonine-protein kinase *At1g05700* in *A. thaliana* (E-value = 1.5 × 10^−89^). Both DEAD-box ATP-dependent RNA helicases and receptor kinases are associated with stress response in Arabidopsis [49,50]. Our analysis indicated that KASP-3 and KASP-6 are non-synonymous mutations resulting in amino acid substitution in *CmoCh20G003040.1* and *CmoCh08G007140.1*, respectively. Taken together, these results suggest altered gene function as a potential cause of phenotype differences between the resistant and susceptible parents. However, further molecular studies are needed to elucidate the functional mechanisms underlying resistance to ZYMV in these candidate genes.

Synteny analysis revealed that QTL blocks on chromosome 20 (*QtlZYMV-C20)* were syntenic with ZYMV resistant genomic regions in cucumber and melon, which suggest a common evolutionary origin of the major ZYMV resistant loci in squash, cucumber and melon.

## 5. Conclusions

QTL-seq method was efficient in identifying QTLs associated with ZYMV resistance in *C. moschata*. The indel and KASP markers developed in the current study will allow rapid discrimination of resistant and susceptible individuals in squash breeding programs. The candidate genes detected should be further investigated to understand the mechanisms underlying ZYMV resistance in squash.

## Figures and Tables

**Table 1:** Whole genome mapping statistics for the parents and bulks used in the study.

Sample	Coverage Rate (%)	Mapping Rate (%)	Sequencing Depth
Nigerian Local	94.42	98.54	85.27
Butterbush	94.51	98.76	101.73
Resistant bulk	91.78	97.29	81.77
Susceptible bulk	92.75	94.04	93.73

**Table 2:** Quantitative trait loci (*p* < 0.05) associated with resistance to Zucchini Yellow Mosaic Virus using either Butterbush or Nigerian Local as the consensus reference genome.

Consensus Reference Genome ^a^	Chromosome	Start (bp)	End (bp)	Length (bp)	Peak ∆ SNP index	Position of Peak SNP (bp)
Butterbush	02	11,491	16,194	4703	0.3523	11,491
	04	9,320,513	10,158,408	837,895	0.3829	9,563,571
	08	3,458,554	5,449,590	1,991,036	0.4147	4,502,562
	20	15,031	2,474,747	2,459,716	0.5244	1,490,841
Nigerian Local	02	9750	24,619	14,869	0.3532	9750
	04	9,416,702	9,870,662	453,960	0.3739	9,563,571
	08	3,653,289	5,446,423	1,793,134	0.4107	4,502,562
	20	17,780	2,474,545	2,456,765	0.5416	1,490,841

^a^ The consensus reference genomes were created by substituting alleles in the published squash reference genome *C. moschata* cv. Rifu with the respective parental alleles.

**Table 3:** List of markers tested on the entire F_2_ population, their chromosomal location and non-parametric Kruskal–Wallis test *p*-values.

Chromosome	Marker	Genomic Location ^1^ (bp)	Kruskal–Wallis *p*-Value
20	Indel-2	1,482,284	0.007 *
20	KASP-1	1,491,656	0.002 *
20	KASP-3	1,486,889	0.006 *
08	KASP-5	4,497,218	0.06
08	KASP-6	4,497,085	0.006 *
04	KASP-10	9,549,004	0.2
02	KASP-14	17,246	0.23

* Significant association of marker with ZYMV resistance (*p* < 0.05). ^1^ Position of marker in *Cucurbita moschata* (Rifu) genome [27].

## Data Availability

Not applicable.

## Supplementary Materials

The following are available online at https://www.mdpi.com/article/10.3390/plants10091935/s1, Table S1: Information on twelve indel and fourteen SNP markers developed from the QTL regions near peak ∆ SNP’s and genotyped in the parents and the resistant and susceptible bulks. Table S2: Result of Kruskal–Wallis test performed using KASP-1, KASP-3 and KASP-6 on BC_1_F_1_ population. Table S3: Information on chromosomal location and putative function of the resistant gene homologs identified within the QTL regions. Figure S1: Quantitative trait loci (QTL) associated with Zucchini Yellow Mosaic Virus resistance in Nigerian Local accession (*Cucurbita moschata*) on chromosome 02, 04, 08 and 20 using Butterbush as consensus reference genome. Red and blue lines represent 95 and 99% confidence interval (CI) respectively. Peaks on or above 95% CI potentially harbor QTLs associated with ZYMV resistance. Figure S2: Quantitative trait loci associated with ZYMV resistance in C. moschata identified on chromosome (chr) 02, 04, 08 and 20 using Butterbush as the parental consensus reference genome. Black line represents tricube smoothed G’ value for each SNP. Red line denotes genome wide false discovery rate (FDR) of 0.01. Peaks above 0.01 FDR line potentially harbor QTLs associated with ZYMV resistance. Figure S3: (a) An agarose gel showing a 245 bp fragment amplified with Indel-2 marker in resistant individuals, but absent in susceptible individuals. (b) Marker assay with KASP-1 (SNP) in a subset of F_2_ individuals segregating for resistance to ZYMV. Green triangles represent resistant individuals carrying the resistance allele, blue triangles represent susceptible individuals carrying the alternative allele and F1’s are represented by red triangles containing both alleles. Gray dots consist of non-template water controls.
