# Supplementary material for: Genetic Loci Associated with Resistance to Zucchini Yellow Mosaic Virus in Squash
Source: Plants (Basel). 2021 Sep 17;10(9):1935. doi: 10.3390/plants10091935 (PMC8465829; doi:10.3390/plants10091935)
Supplement: Supplementary file 1 [file plants-10-01935-s001.zip › plants-1349697-Figure S1.pdf]

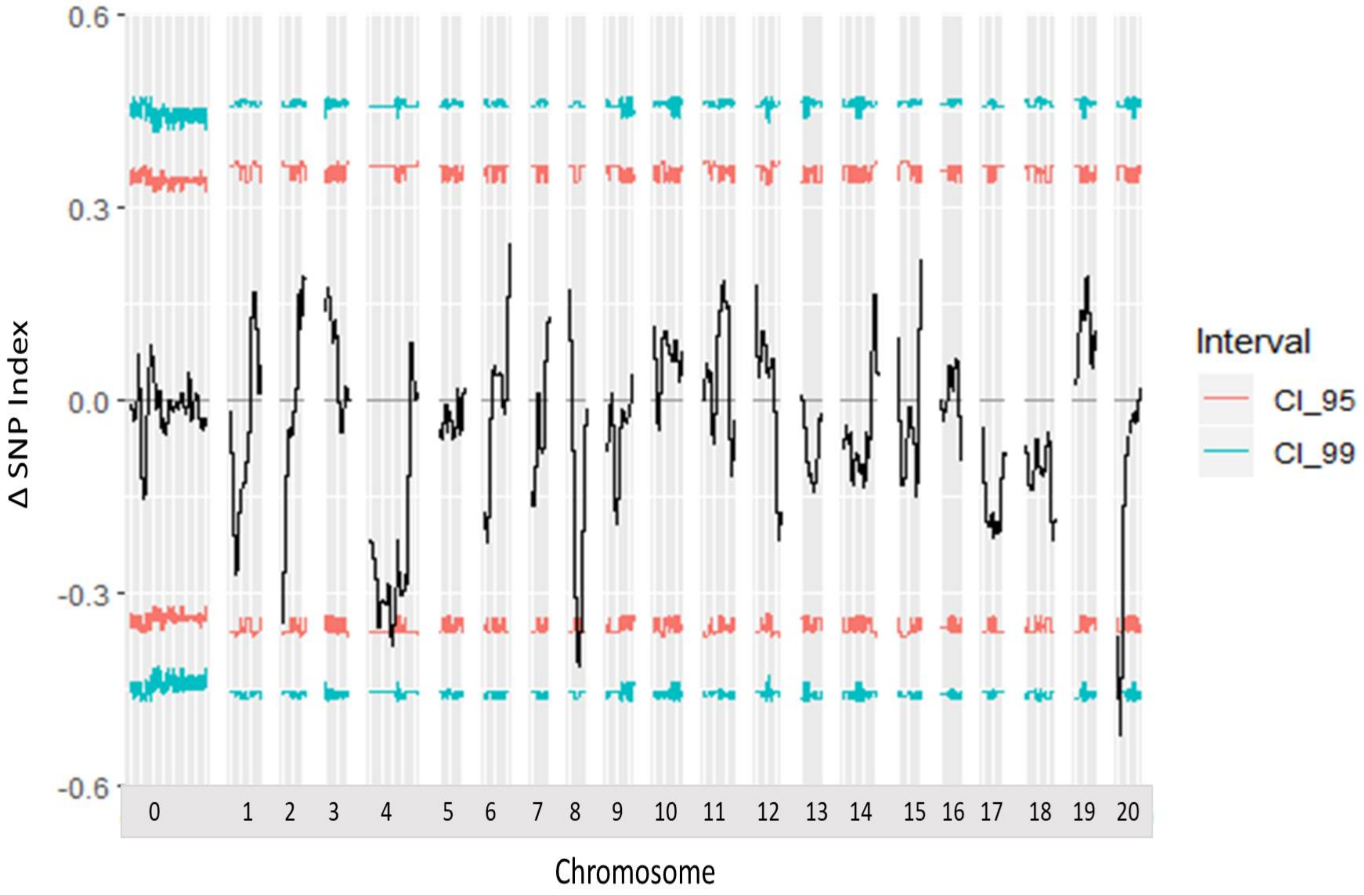

Figure S1: Quantitative trait loci (QTL) associated with Zucchini Yellow Mosaic Virus resistance in Nigerian Local accession (*Cucurbita moschata*) on chromosome 02, 04, 08 and 20 using Butterbush as consensus reference genome. Red and blue lines represent 95 and 99 % confidence interval (CI) respectively. Peaks on or above 95 % CI potentially harbor QTLs associated with ZYMV resistance.
