# Supplementary material for: Genetic Loci Associated with Resistance to Zucchini Yellow Mosaic Virus in Squash
Source: Plants (Basel). 2021 Sep 17;10(9):1935. doi: 10.3390/plants10091935 (PMC8465829; doi:10.3390/plants10091935)
Supplement: Supplementary file 1 [file plants-10-01935-s001.zip › plants-1349697-Figure S2.pdf]

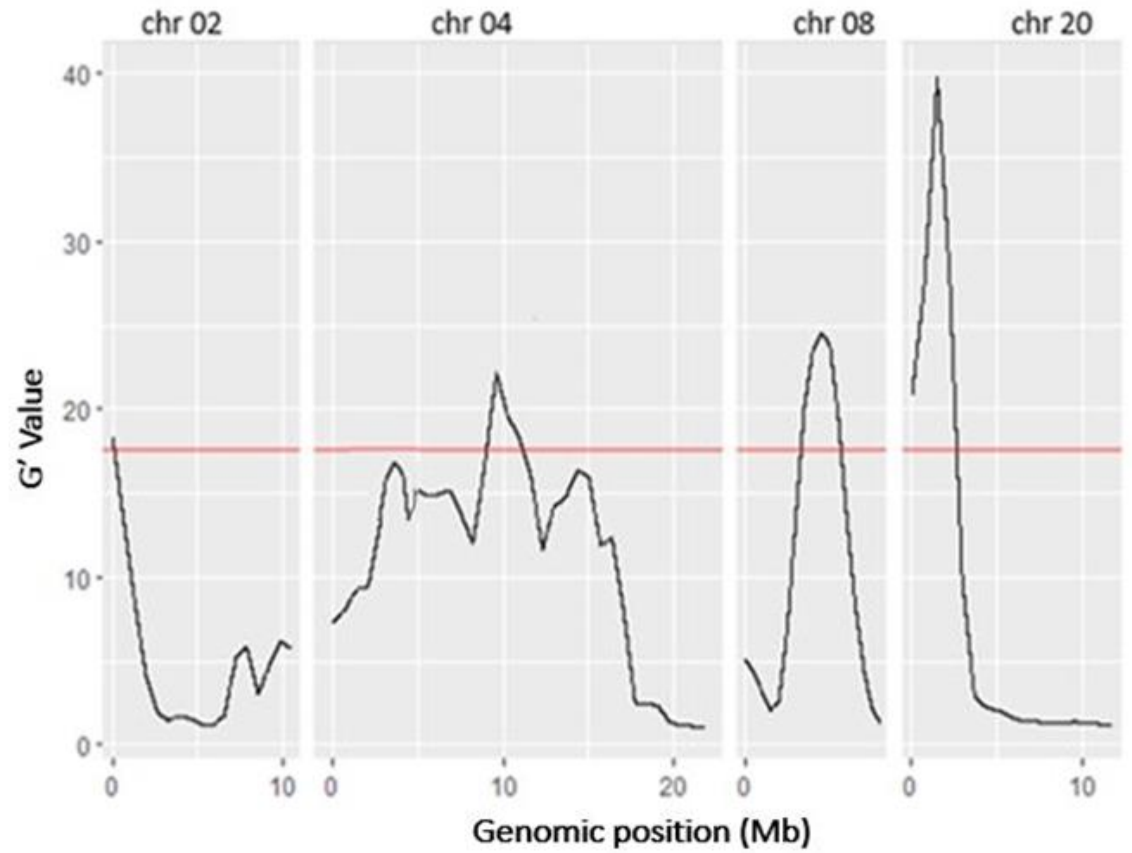

Figure S2: Quantitative trait loci (QTL) associated with ZYMV resistance in *C. moschata* identified on chromosome (chr) 02, 04, 08 and 20 using Butterbush as the parental consensus reference genome. Black line represents tricube smoothed G' value for each SNP. Red line denotes genome wide false discovery rate (FDR) of 0.01. Peaks above 0.01 FDR line potentially harbor QTLs associated with ZYMV resistance.
