# Supplementary material for: Genetic Loci Associated with Resistance to Zucchini Yellow Mosaic Virus in Squash
Source: Plants (Basel). 2021 Sep 17;10(9):1935. doi: 10.3390/plants10091935 (PMC8465829; doi:10.3390/plants10091935)
Supplement: Supplementary file 1 [file plants-10-01935-s001.zip › plants-1349697-Figure S3.pdf]

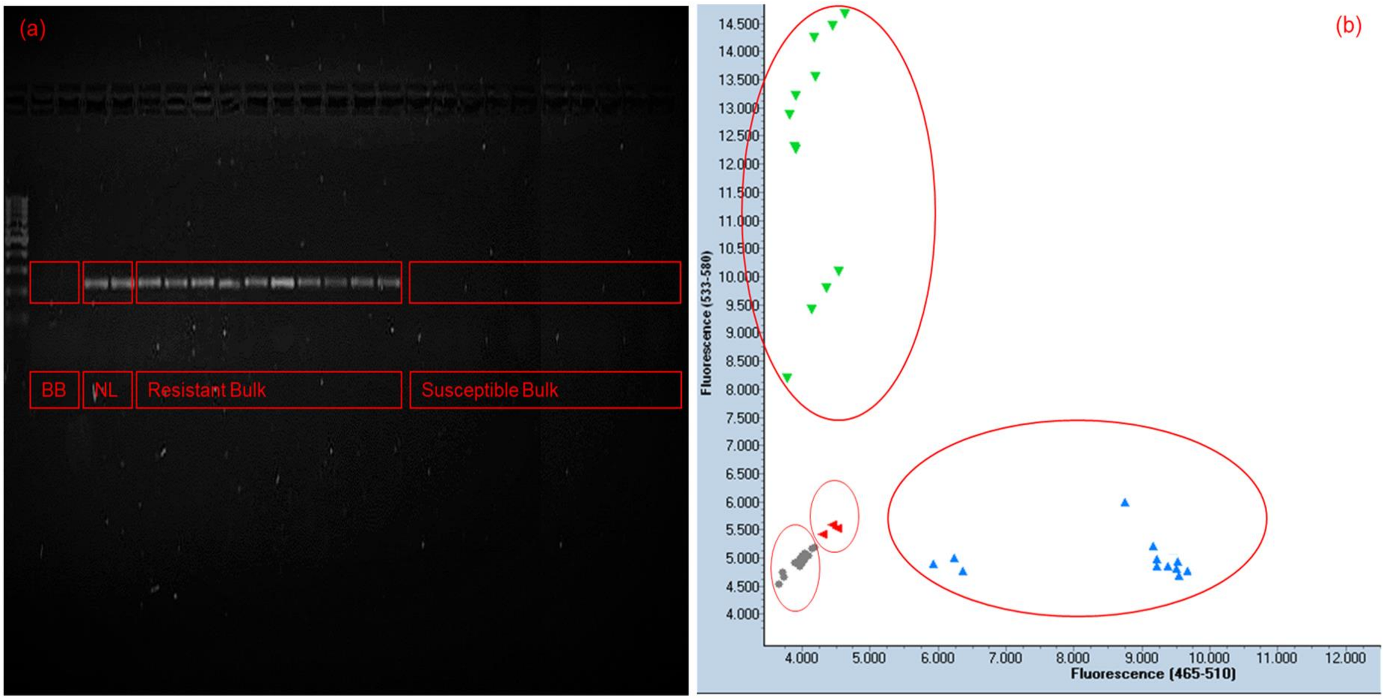


Figure S3: (a) An agarose gel showing a 245 bp fragment amplified with Indel-2 marker in resistant individuals, but absent in susceptible individuals. (b) Marker assay with KASP-1 (SNP) in a subset of F2 individuals segregating for resistance to ZYMV. Green triangles represent resistant individuals carrying the resistance allele, blue triangles represent susceptible individuals carrying the alternative allele and F1's are represented by red triangles containing both alleles. Gray dots consist of non-template water controls.
